# Using the latent diffusion model to enhance time-resolved laser speckle contrast imaging (TR-LSCI) of cerebral blood flow

**DOI:** 10.1364/BOE.567377

**Published:** 2025-09-02

**Authors:** Faraneh Fathi, Rabeya Tus Sadia, Mehrana Mohtasebi, Paul Mos, Claudio Bruschini, Edoardo Charbon, Lei Chen, Jin Chen, Guoqiang Yu

**Affiliations:** 1 Department of Biomedical Engineering, University of Kentucky, Lexington, Kentucky, 40506, USA; 2 Department of Computer Science, University of Kentucky, Lexington, Kentucky, 40506, USA; 3 Bioptics Technology LLC, Advanced Science & Technology Commercialization Center (ASTeCC), Lexington, Kentucky, USA; 4 School of Engineering, Ecole polytechnique fédérale de Lausanne, Neuchâtel 2002, Switzerland; 5 Department of Neurosurgery, University of Kentucky, Lexington, Kentucky, 40536, USA; 6 Department of Biomedical Informatics and Data Science, University of Alabama at Birmingham, Birmingham, Alabama, 35294, USA

## Abstract

Continuous monitoring of cerebral blood flow (CBF) with high spatiotemporal resolution and depth sensitivity is essential for accurate diagnosis and effective management of neurological disorders. Although conventional laser speckle contrast imaging (LSCI) enables widefield, high-resolution CBF mapping, its limited penetration depth and signal integration across all tissue layers hinder depth-resolved imaging. To address these limitations, we developed an advanced time-resolved LSCI (TR-LSCI) system that employs picosecond-pulsed laser illumination and a customized SPAD512^2^ camera operating in gated mode, enabling noncontact, widefield, and depth-sensitive CBF imaging. However, photon scattering and diffusive noise still degrade image quality, particularly at greater depths. To overcome this challenge, we incorporated a multiscale latent diffusion model (LTDiff++) into the TR-LSCI analysis pipeline to suppress photon diffusion noise. Trained and validated using overlapping image patches from head-simulating phantoms and neonatal rat CBF images with high-quality ground truth references, LTDiff++ effectively suppressed photon diffusion noise while preserving structural and vascular features at greater imaging depths. Moreover, in vivo studies demonstrated that LTDiff++ maintained image quality using only 5-frame averaging, reducing acquisition time by a factor of 20 compared to the conventional 100-frame averaging approach without deep learning enhancement. The integrated TR-LSCI and LTDiff++ framework thus enables robust, high-speed, and depth-resolved imaging of cerebral hemodynamics, offering a promising platform for preclinical research and future clinical applications in bedside neuroimaging and patient monitoring.

## 1. Introduction

The human brain, despite comprising only 2% of total body weight, accounts for nearly 20% of total oxygen and energy consumption, underscoring its exceptionally high metabolic demand [1]. This demand is met through cerebral blood flow (CBF), which ensures a continuous supply of oxygen and nutrients while facilitating the removal of metabolic waste. To maintain optimal blood perfusion despite fluctuations in systemic blood pressure, CBF is tightly regulated by a complex interplay of physiological mechanisms, including autoregulation, neurovascular coupling, and endothelial function [2–4]. Given its fundamental role in brain function and pathology, accurate and continuous measurement of CBF is essential for both the diagnosis and management of neurological diseases.

A variety of large imaging techniques have been developed to assess CBF, including arterial spin labeling magnetic resonance imaging (ASL-MRI) [5,6], dynamic computed tomography (CT) [7], and positron emission tomography (PET) [8,9]. Although these imaging techniques offer crucial insights into cerebral hemodynamics, their high cost, low sampling rate, electromagnetic compatibility constraints, lack of portability, radiation exposure, and use of ionizing radiation (CT and PET) limit their utility for real-time, continuous, and longitudinal bedside CBF monitoring. In contrast, optical imaging modalities provide a noninvasive, cost-effective, and portable alternative, enabling real-time monitoring of CBF dynamics with high spatiotemporal resolution.

Conventional laser speckle contrast imaging (LSCI) uses continuous-wave (CW), widefield, coherence illumination and employs ordinary charge-coupled device/complementary-metal-oxide-semiconductor (CCD/CMOS) cameras to capture laser speckle contrast fluctuations [10,11]. LSCI measures CBF by analyzing the temporal and spatial fluctuations of laser speckle patterns generated when coherent laser light scatters off moving red blood cells. In a static medium, the speckle pattern remains unchanged during the camera's exposure time, resulting in high speckle contrasts. In contrast, when scatterers such as red blood cells are moving, the speckle pattern changes dynamically, causing a reduction in speckle contrasts. The faster the blood flow, the more rapidly the speckle pattern decorrelates during the exposure time, leading to lower speckle contrasts [12]. LSCI enables two-dimensional (2D) high-resolution mapping of CBF but is restricted to superficial cortical regions due to its shallow penetration depth (<1 mm), necessitating invasive procedures for deeper brain imaging [13,14]. Additionally, LSCI collects signals from all depths, which compromises depth sensitivity and limits its ability to distinguish between superficial and deep tissue blood flow.

To overcome the limitations of existing technologies, we have recently developed a novel time-resolved laser speckle contrast imaging (TR-LSCI) system that enables noncontact, widefield, and depth-sensitive imaging of CBF [15–17]. In contrast to CW-LSCI, TR-LSCI utilizes picosecond-pulsed, widefield, coherent near-infrared illumination and synchronizes a picosecond-gated, single-photon-avalanche-diode (SPAD) camera to detect photons traveling through deeper tissue layers. By employing the time-gating strategy, TR-LSCI differentiates short and long photon paths, allowing for depth-resolved imaging without the need for complex tomographic reconstruction. This advancement enables fast, high-resolution, 2D mapping of CBF over a large region-of-interest (ROI) while maintaining the necessary spatiotemporal resolution to capture rapid cerebral hemodynamic changes. We have demonstrated the depth sensitivity of TR-LSCI for continuous imaging of CBF at different depths through extensive experiments using head-simulating phantoms and rodent models [16].

Despite the advancement of TR-LSCI, CBF imaging faces challenges in suppressing diffusive noises, particularly in deeper tissue regions where photon scattering significantly degrades signal fidelity. In this project, we incorporated a multiscale latent diffusion model (LTDiff++) into TR-LSCI data analysis, integrating a UNet++ encoder-decoder network with a denoising diffusion probabilistic model (DDPM) to improve depth sensitivity and enhance image quality. DDPM is a generative deep-learning framework that iteratively refines noisy input data through a learned stochastic diffusion process [18–21], effectively improving the robustness and quality of the reconstructed images. DDPM has been successfully applied to various medical imaging modalities, including MRI, CT, and PET, for tasks such as image classification, segmentation, reconstruction, and noise suppression [22–25]. Compared to generative adversarial networks and variational auto-encoders, DDPM shows superior performance in image generation.

Inspired by these advancements, we implemented LTDiff++ for TR-LSCI analysis to tackle challenges of photon scattering and diffusive noises in depth-sensitive CBF imaging. We evaluated its performance using head-simulating phantoms and in vivo neonatal rat models, demonstrating enhanced imaging robustness against diffusive noises and improved image quality. Consequently, this approach improved spatial resolution and shortened acquisition time, enabling TR-LSCI to achieve greater depth sensitivity while preserving its noncontact, continuous, and widefield CBF imaging capability.

## 2. Methods and materials

### 2.1. TR-LSCI prototype and optimization

The TR-LSCI prototype, as reported in our previous paper [16], was initially developed using a picosecond-pulsed laser (775 nm, Katana-08 HP, NKT Photonics) and a customized SwissSPAD2 camera (EPFL, Switzerland), synchronized at 20 MHz. Although the SwissSPAD2 camera supports a resolution of 512 × 512 pixels with the integration of an external FPGA, our previous study utilized a reduced resolution of 512 × 256 pixels without additional hardware [16]. Data acquisition with SwissSPAD2 required a two-step workflow involving a 32-bit MATLAB script for acquisition and a separate 64-bit script for converting binary frames to TIFF format, introducing unnecessary complexity and reducing user-friendliness.

To enhance performance and operational efficiency, the TR-LSCI platform was upgraded with a customized SPAD512^2^ (Pi Imaging, Switzerland) camera, replacing the SwissSPAD2. The SPAD512^2^ offers a full 512 × 512 pixel array, effectively doubling the spatial resolution compared to the 512 × 256 configuration previously used with the SwissSPAD2. Moreover, the SPAD512^2^ camera operates with an optimized C++ based acquisition system that directly generates PNG images in real-time, significantly simplifying data handling and improving operational efficiency. In addition, the SPAD512^2^ comes with a user-friendly software package that significantly simplifies system control and operation. Specifically, the SPAD512^2^ features a graphical user interface (GUI) and remote command interface for precise control of acquisition parameters like exposure time, gate steps, gate step size, gate width, and offset time. Dedicated tabs for “Live View,” “Intensity Imaging,” “Gated Imaging,” and “Settings” enhance usability, with controls on the left and real-time images on the right for immediate feedback.

Enhancements to SPAD512^2^ hardware and software have significantly advanced TR-LSCI’s capability for real-time, high-resolution, and depth-resolved CBF imaging. The transition from SwissSPAD2 to SPAD512^2^ has ultimately produced a user-friendly, high-performance, and more compact TR-LSCI system.

**Fig. 1.**
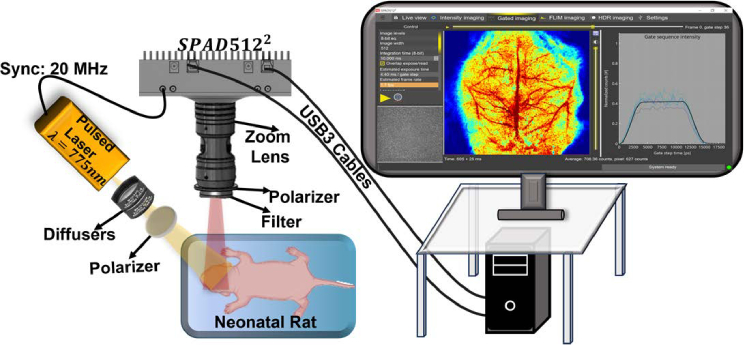
**Upgraded TR-LSCI system for 2D imaging of CBF distributions at different depths in neonatal rats.** The system consists of a picosecond-pulsed laser at 775 nm and a high-performance SPAD512^2^ camera, both supported on an optical table. The pulsed laser operates at 20 MHz, producing synchronization triggers for the camera to capture gated images at different depths. Key components of the TR-LSCI system include a picosecond-pulsed laser to generate collimated coherent light, two optical diffusers to create the widefield illumination, a gated SPAD512^2^ camera to capture spatial speckle contrast images, a zoom lens to adjust the ROI, a long-pass filter to minimize ambient light impact, and a pair of polarizers across the source and detection paths to minimize tissue surface reflection.

The upgraded TR-LSCI system remains a benchtop, noncontact imaging setup for continuous monitoring of CBF variations in rodents (Fig. 1). Coherent illumination is provided by the same picosecond-pulsed laser at 775 nm (Katana-08 HP), with the utilization of two engineered optical diffusers (ED1-C20-MD and ED1-S20-MD, Thorlabs) ensuring widefield illumination. A zoom lens (Zoom 7000, Navitar) was attached to the SPAD512^2^ camera to adjust the ROI, set to an F number of 11 to satisfy the Nyquist criterion. A long-pass filter (>750 nm, 84–761, Edmund Optics) was used to minimize ambient light impact. Two crossed polarizers (LPNIRE050-B and LPNIRE200-B, Thorlabs) were included to the source and detection paths respectively to minimize light reflection from the tissue surface. Data transfer was achieved via a USB cable from the FPGA board on the camera to a dedicated computer as a control unit.

### 2.2. TR-LSCI principle, data acquisition, and data analysis

The pulsed laser and SPAD camera are synchronized at 20 MHz, resulting in a 50 ns period. The camera operates in gated mode, where the time gating functions as a filter, capturing photons only when they arrive within a specific time window. By systematically shifting the gate window with a minimal step of ∼18 ps, the system captures photons scattered from various depths, allowing for depth-resolved CBF imaging.

Overlapping gates were created when the gate step size (delay between adjacent gate positions) was smaller than the camera gate width. This overlapping gating mechanism, combined with the ∼18 ps gate step size, enables sub-nanosecond temporal resolution. The laser pulse acts as a timing reference for the camera’s gated detection mechanism. The camera gate is triggered after a specific delay (t_0_ + Δt), where t_0_ is the initial offset time, and Δt is the incremental gate step, which is fixed at ∼18 ps. After each gated acquisition, Δt is progressively increased, shifting the gate window step by step. This sequential adjustment allows the system to capture photons arriving at different time delays, enabling depth-sensitive CBF imaging.

The data processing pipeline for TR-LSCI follows the approach outlined in our previous publication [16]. For speckle contrast analysis, raw intensity images captured by the SPAD camera are directly processed using LSCI analysis: the speckle contrast $\left(K_{s}\right)$ is calculated as: Ks=σ
s⟨I⟩=⟨I2⟩−
<I>2⟨I⟩ , where σ
s is the standard deviation of intensity and ⟨
I⟩
 is the mean intensity within a 3 × 3 pixel window. The blood flow index (BFI) is then estimated using the standard inverse square relationship: $BFI∼\frac{1}{K_{s}^{2}}$ .

In the phantom experiment, TR-LSCI measurements were acquired using the SwissSPAD2 camera with a 9.38 ms exposure time per 10-bit image and a gate width of 23 ns. Data acquisition included 200 gate steps with an offset time of 30.5 ns and a gate step size of ∼18 ps, resulting in a total detection window of 3.6 ns.

In the animal experiment, TR-LSCI measurements were acquired using the SPAD512^2^ camera with a 2.6 ms exposure time per 10-bit image and a gate width of 13 ns. A total of 80 gate steps were collected with an offset time of 49.48 ns and a gate step size of ∼18 ps, resulting in an overall detection window of 1.4 ns. In our previous publication [16], we established that each gate delay of 18 ps corresponds to a distance increment of approximately 63 µm, reflecting photon travel distance rather than direct imaging depth. In general, gate positions used in this study correspond to imaging depths of approximately 1.5 to 3 mm in layered scattering media.

The new SPAD512^2^ camera incorporates built-in noise and dead pixel corrections, eliminating the need for additional post-processing steps required by the SwissSPAD2 camera. These corrections, including noise correction, pile-up correction, and dead pixel interpolation, are directly managed by the SPAD512^2^ software, ensuring more accurate and linearized intensity measurements. This enhancement significantly reduces computational overhead and improves the accuracy of depth-resolved CBF mapping.

### 2.3. LTDiff++ model for image enhancement

**Fig. 2.**
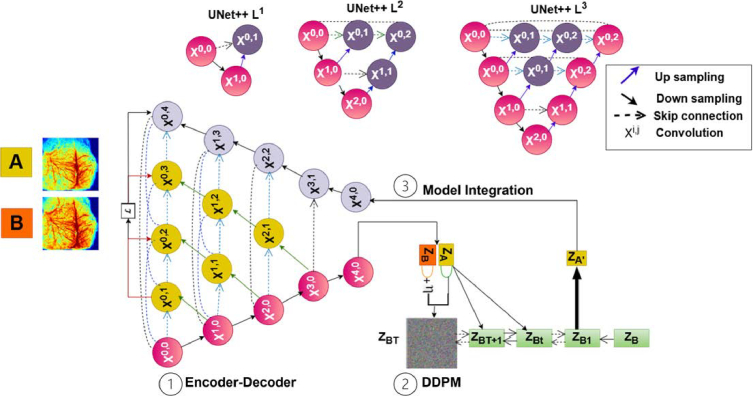
**Overview of LTDiff++ architecture for image enhancement.** The model integrates a UNet++ encoder-decoder for hierarchical feature extraction and a conditional DDPM for latent-space denoising. Given an image pair (A, B), where A is a training input image and B is its corresponding ground truth, the model aims to synthesize a standardized image A’ that aligns with domain B. In this process, Z_A_ and Z_B_ represent the latent space embeddings of A and B, respectively. This framework is pivotal for learning encoded latent representations of TR-LSCI images. The diffusion process introduces Gaussian noise (η) to Z_A_, followed by iterative denoising using information from Z_B_. This framework refines brain vascular structures while minimizing noise and artifacts, improving the clarity and consistency of diffuse speckle-based imaging.

In deep learning, a latent space is a lower-dimensional embedding of input images, where important patterns, textures, and structures are preserved, but noise and redundant information are filtered out. This makes the latent space an efficient domain for operations like noise reduction, feature refinement, and image standardization [26]. In this study, a multiscale latent diffusion model (LTDiff++) was employed to reduce photon diffusive noises in TR-LSCI images. The LTDiff++ architecture shown in Fig. 2 consists of two primary components: **(1)** A UNet++ encoder-decoder network, which extracts hierarchical features from input images and generates a compact latent representation. **(2)** A conditional DDPM, which refines this latent representation by progressively removing noise and aligning the output with the ground truth distribution. DDPM learns a Markov chain to gradually convert a simple distribution, such as isotropic Gaussian, into a desired data distribution [20,21].

In Fig. 2, A and B represent the training-input and ground-truth images, respectively, while Z_A_ and Z_B_ denote their corresponding latent representations. Once the latent space is constructed, the conditional DDPM operates within this domain to model the probability distribution of the latent representations. The diffusion process begins with controlled Gaussian noise (η) added to Z_A_, simulating a forward diffusion step. The reverse diffusion model then progressively denoises Z_A_ using information from Z_B_ to align the output with the ground truth image distribution. This approach minimizes photon diffusive noises and preserves fine structural details, significantly enhancing spatial resolution and shortening acquisition time.

Model training follows a three-phase approach aligned with the model architecture: **(1)** **Encoder-Decoder Training**. The encoder-decoder network is first trained independently using a diverse set of images irrespective of whether they are training input or ground truth to minimize reconstruction error and ensure effective translation into 1D latent vectors while preserving structural details. The UNet++ architecture includes skip connections between the encoder and decoder to maintain high-resolution information and improve feature extraction, particularly for subtle vascular details. **(2)** **DDPM Training**. The conditional DDPM is then trained using paired training-input and ground-truth images, learning the conditional probability distribution of latent representations. DDPM employs a stochastic diffusion process where controlled Gaussian noise is added to the latent space representation, and a reverse diffusion process iteratively denoises the representation to match the target image distribution. This phase enables the DDPM to progressively refine the latent space and enhance the quality of the output images. **(3) Model Integration**. After training the encoder-decoder and DDPM separately, the components are integrated into a unified model. The complete model processes an input image through the encoder to generate a latent representation, refines this representation through the DDPM, and reconstructs the final enhanced image through the decoder. The integrated framework allows LTDiff++ to generate standardized and high-fidelity images from newly acquired input data with improved noise suppression and spatial consistency. For training, an Adam optimizer was used with a learning rate of 0.0001, a batch size of 45, and 500 epochs to ensure stable convergence.

### 2.4. Data preprocessing, augmentation, and evaluation metrics for LTDiff++ modeling

Before training, TR-LSCI data undergoes preprocessing to optimize learning. The input image and its corresponding ground truth are divided into multiple overlapping patches, each measuring 64 × 64 pixels. This approach ensures that the model learns localized features effectively while preserving global spatial consistency across images.

**Fig. 3.**
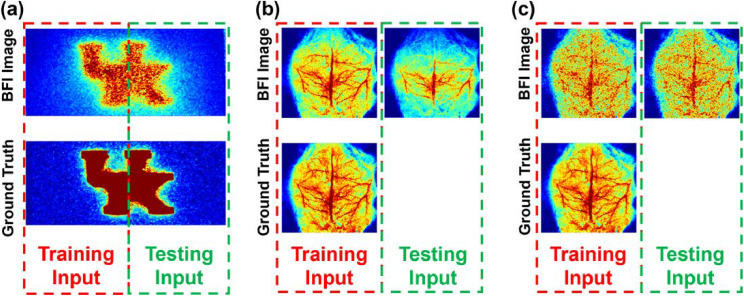
**Data partitioning and preprocessing for the head-simulating phantoms and rats. (a)** The BFI image patches extracted from the left side of the phantom image (red box) were used to train the LTDiff++ model, while patches from the right side (green box) were used to test the model. The ground truth BFI image was taken from gate #0 of the same UK logo phantom, flipped upside down. **(b)** To improve spatial resolution, BFI images of a neonatal rat from Gates #40 and #50, each generated by averaging 100 time-course BFI images, were used for training and testing, respectively. **(c)** To improve temporal resolution, BFI images from Gates #0 and #1, each generated by averaging 5 time-course images, were used for training and testing, respectively. For both scenarios of (b) and (c), the average BFI image at Gate #0 (obtained by averaging 100 time-course images) was used as the ground truth, ensuring a consistent reference for model training and evaluation.

**Fig. 4.**
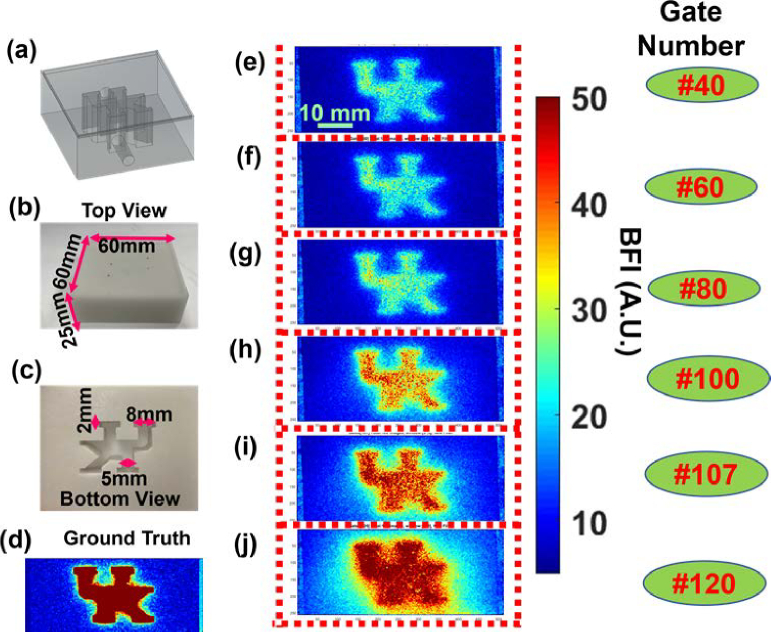
**Depth-sensitive TR-LSCI measurement of 3D-printed head-simulating phantom. (a)-(c)** The top and bottom views of a UK logo solid phantom with a top layer thickness of 1 mm. The empty UK letter channels were filled with a liquid phantom solution containing Intralipid particles to simulate flow contrast. **(d)** The ground-truth BFI image taken at Gate #0 from a flipped-over UK logo phantom (c). **(e)-(j)** Reconstructed BFI maps obtained using the TR-LSCI at the gates of #40, #60, #80, #100, #107, and #120, demonstrating its depth sensitivity. To enhance SNR, 100 time-course images were captured and averaged at each gate. The imaging parameters include a pulse laser frequency of 20 MHz (50 ns period), an offset time of 30.5 ns, a gate delay time of 18 ps, and a total of 200 gates, resulting in a detection time window of 3.6 ns.

For the phantom data (Fig. 3(a)), an overlapping patch-based strategy was applied to segment the BFI dataset into training and testing regions. The left part of the University of Kentucky (UK) logo was used for model training, generating 1,394 image patches of 64 × 64 pixels with an 80% overlap. The right part of the UK logo was designated for testing, producing 21,896 patches with a 95% overlap. This partitioning ensured that the model was trained on representative features while being evaluated on unseen regions. The ground truth BFI image was taken from Gate #0 of the same UK logo phantom, flipped upside down (Fig. 4(d)).

For the animal data, BFI images obtained from the entire neonatal rat head at different gates were used for training and testing without splitting them into left and right regions. In one scenario aimed at enhancing spatial resolution at greater depths, the BFI image acquired at Gate #40 was used for training, while the BFI image at Gate #50 was used for testing (Fig. 3(b)). Both images were generated by averaging 100 time-course BFI frames. This resulted in 26,208 patches of 64 × 64 pixels with a 95% overlap for both training and testing. In another scenario aimed at shortening acquisition time, BFI images with reduced number of averages were used as inputs (Fig. 3(c)). Specifically, the BFI image at Gate #0 was used for training, and the BFI image at Gate #1 for testing, with both generated by averaging 5 time-course BFI images while maintaining the same patching strategy. For both scenarios, the average BFI image at Gate #0, derived from 100 time-course images, served as the ground truth, providing a consistent reference for model training and evaluation.

Data augmentation techniques including random horizontal flipping and small-angle rotations (±10 degrees) were applied on the data from both phantoms and animals to improve generalization and introducing variability to the dataset. The preprocessed patches were then processed through a two-stage training pipeline (Fig. 2).

The performance of the LTDiff++ model was assessed using peak signal to noise ratio (PSNR) and structural similarity index measure (SSIM) between two images. PSNR was calculated as: $PSNR=10\log_{10}\left(\frac{MAX^{2}}{MSE}\right)$ , where MAX is the maximum possible BFI value in the ground truth image, and MSE represents the mean squared error between the ground truth image and reconstructed flow images at different gates. A higher PSNR indicates better reconstruction quality. The SSIM evaluates the perceptual similarity between two images by considering luminance, contrast, and structural differences. Unlike PSNR, which measures absolute errors, SSIM provides a more human-perceptible assessment of image quality. SSIM is calculated as: SSIM(x,y)=(2μ
xμ
y+C1)(2σ
xy+C2)(μ
x2+μ
y2+C1)(σ
x2+σ
y2+C2) , where μ
x and μ
y are the mean intensities, σ
x and σ
y are the standard deviations, and σ
xy is the covariance of the two images. The constants $C_{1}$ and $C_{2}$ stabilize the division.

### 2.5. Phantom experimental protocols

A head-simulating phantom with well-defined optical properties and geometry (Fig. 4(a)–4(c)) was fabricated by a 3D printer (SL1, Prusa) to evaluate the depth sensitivity of TR-LSCI. Specifically, a solid phantom with a top layer thickness of 1 mm was fabricated, as it closely approximates the skull thickness of neonatal rats. The 3D-printed solid phantom composed of titanium dioxide (TiO_2_), India ink (Black India, MA), and clear resin (eSUN Hard-Tough) to replicate the scattering and absorption characteristics of biological tissue. The embedded UK logo-shaped channel was filled with a liquid phantom solution containing intralipid particles (Fresenius Kabi, Sweden), India ink, and water. India ink concentration controlled the absorption coefficient (μ_a_), and TiO_2_ and Intralipid concentrations determined the reduced scattering coefficient (μ_s_’). The Brownian motion of Intralipid particles mimicked the movement of red blood cells in brain tissue. The optical properties of both the solid and liquid phantom were set as: μ_a_ = 0.03 cm^−1^ and μ_s_’ = 9 cm^−1^.

The old TR-LSCI system, equipped with SwissSPAD2 camera, was used to image a ROI of 30 × 60 mm^2^ on the phantom surface. A total of 200 gated images were acquired at sequential depths with an ∼18 ps delay per gate.

### 2.6. In vivo experimental protocols

All animal procedures were approved by the UK Institutional Animal Care and Use Committee (IACUC). Two neonatal rats (7 and 8 days postpartum) were studied. Each rat was anesthetized using Isoflurane (1-2%) and placed on a heating blanket to maintain body temperature. The head was stabilized in a stereotaxic frame for TR-LSCI measurements. To minimize partial volume effects on deep brain imaging, the mouse scalp was surgically retracted.

The upgraded TR-LSCI system, equipped with SPAD512^2^ camera, was used to image a ROI of 17 × 17 mm^2^ on the head. To balance depth sensitivity and temporal resolution, 80 gated images were acquired with a gate delay of ∼18 ps. This approach enabled a higher temporal sampling rate compared to the phantom experiments, which utilized 200 gated images.

## 3. Results

### 3.1. TR-LSCI enables depth-sensitive imaging of particle flow in head-simulating phantoms

Figure 4(e)–4(j) present 2D flow contrast maps of the head-simulating phantom obtained by the TR-LSCI at the gates of #40, #60, #80, #100, #107, and #120. At each gate, 100 time-course images were acquired and averaged to enhance the SNR. The results demonstrate a progressive increase in flow contrast with deeper gate positions, verifying the depth-sensitive capability of TR-LSCI. However, as the gate number increased (from #40 to #120), the SNR decreased due to reduced photon detection and elevated diffusive noise at greater depths.

### 3.2. LTDiff++ Enhances TR-LSCI images of head-simulating phantoms by reducing diffusive noise and improving structural integrity

**Fig. 5.**
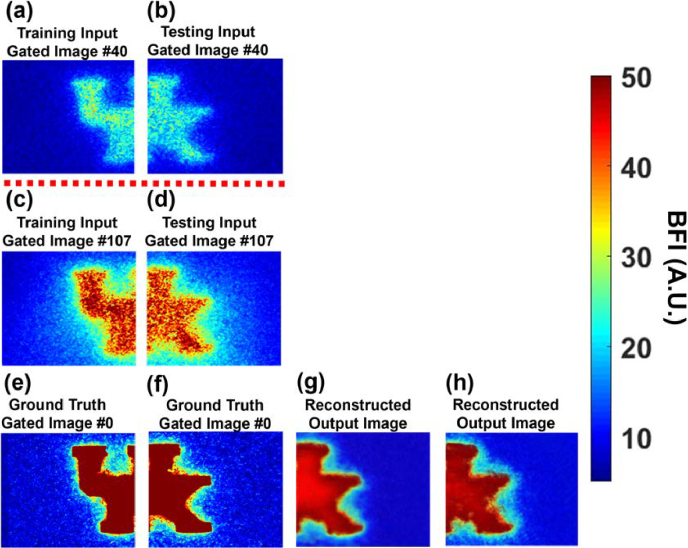
**BFI image enhancement of UK logo phantoms by the LTDiff++ model. (a)** and **(b)** The BFI image patches taken at Gate #40 from the UK logo phantom with a top layer thickness of 1 mm. **(c)** and **(d)** Input BFI image patches at Gate #107 from the same phantom. The BFI image patches from the left and right sides of the phantom were used for training and testing the LTDiff++ model, respectively. **(e)** and **(f)** The ground-truth BFI images taken at Gate #0 from a flipped-over UK logo phantom. **(g)** and **(h)** The resulting BFI images at Gate #40 and Gate #107 preserve the structural information and contains less noise than their input images of (b) and (d).

Figure 5 presents the results of image enhancement using the LTDiff++ model for UK logo phantoms acquired at two representative gate positions: Gate #40 and Gate #107. These gates were selected to evaluate the model's denoising performance at different depths, with Gate #40 representing a mid-range depth and Gate #107 exhibiting increased photon diffusive noise at greater depth. The input and ground truth BFI images were obtained using the SwissSPAD2 camera. Figure 5(a) and Fig. 5(b) show input BFI images at Gate #40 used for training and testing, respectively. Figure 5(c) and Fig. 5(d) display input BFI images at Gate #107 used for training and testing. Figure 5(e) and Fig. 5(f) show the ground truth BFI images taken from Gate #0 of the UK logo phantom, flipped upside down (Fig. 4(d)). Figure 5(g) and Fig. 5(h) present the resulting model output BFI images for Gate #40 and Gate #107 respectively, which preserves structural information and contains less noise than their respective input images (Fig. 5(b) and Fig. 5(d)).

The results were evaluated using PSNR and SSIM (Section 2.4). Compared to the input images at Gate #40 (Fig. 5(b)) and Gate #107 (Fig. 5(d)), the synthesized outputs (Fig. 5(g) and Fig. 5(h) showed significant improvements in both PSNR and SSIM. For Gate #40, PSNR increased from 7.04 to 15.61 dB, and SSIM improved from 0.31 to 0.36. For Gate #107, PSNR improved from 9.77 to 15.03 dB, and SSIM increased from 0.31 to 0.35. All measurements were computed with respect to the ground truth (Fig. 5(e) and Fig. 5(f)). Higher SSIM values indicate greater structural similarity to the reference image. These results demonstrate the effectiveness of LTDiff++ in mitigating photon diffusive noises while preserving structural details of the UK logo phantom.

### 3.3. TR-LSCI enables depth-sensitive imaging of cerebral vasculature in neonatal rats

**Fig. 6.**
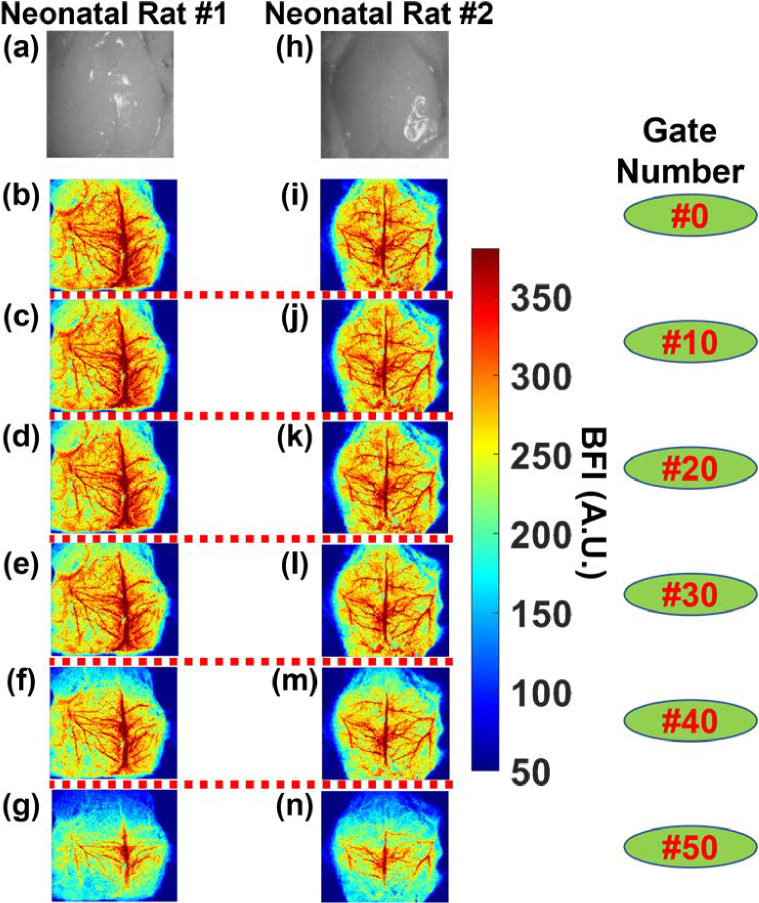
**Depth-sensitive TR-LSCI imaging of CBF in neonatal rats. (a)** and **(h)** The white light images of two neonatal rat heads with their nose up and scalp retracted. **(b)-(g)** and **(i)-(n)** The reconstructed BFI maps obtained by the TR-LSCI at the gates of #0, #10, #20, #30, #40, and #50 for Rat #1 and Rat #2, respectively, demonstrating the progressive loss of vascular visibility with increasing gate number. To enhance SNR, 100 time-course images were captured and averaged at each gate. The imaging parameters include a laser frequency of 20 MHz (50 ns period), an offset time of 49.48 ns, a gate delay time of ∼18 ps, and a total of 80 gates, resulting in a detection time window of 1.4 ns.

To evaluate the depth sensitivity of TR-LSCI in vivo, two neonatal rats were imaged by the SPAD512^2^ camera at 80 gated positions with a gate step size of ∼18 ps. Figure 6(a) and Fig. 6(h) show white light images of two neonatal rats with their scalp retracted. Figure 6(b)–6(g) and Fig. 6(i)–6(n) present BFI maps at selected gates of #0, #10, #20, #30, #40, and #50 for neonatal Rat #1 and Rat #2 respectively, illustrating the variations in visibility of cerebral vasculature as the gate number increases. At each gate, 100 time-course images were acquired and averaged to improve the SNR. At lower gate numbers (e.g., Gate #0), superficial vascular structures are clearly resolved with high contrast, while deeper vessels appear less distinct (e.g., Gate #50). As the gate number increases, the visibility of vascular details diminishes, consistent with the expected attenuation of photons scattered from deeper tissue layers.

### 3.4. LTDiff++ Enhances spatial resolution of TR-LSCI for CBF images in neonatal rats

**Fig. 7.**
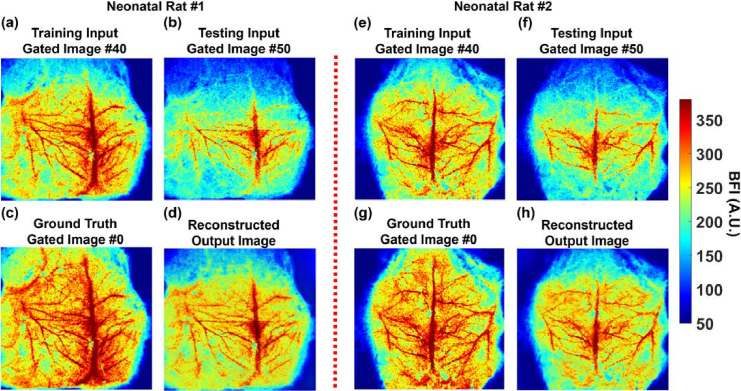
**Spatial resolution enhancement of CBF images in two neonatal rats by the LTDiff++ model. (a)** and **(e)** The average BFI image patches taken at Gate #40 for training the LTDiff++ model in Rat #1 and Rat #2, respectively. **(b)** and **(f)** The average BFI image patches taken at Gate #50 for testing the LTDiff++ model in Rat #1 and Rat #2, respectively. **(c)** and **(g)** The average ground truth BFI images taken at Gate #0 for Rat #1 and Rat #2, respectively All BFI images were obtained by averaging 100 time-course images to improve SNR. **(d)** and **(h)** The resulting output BFI images at deeper depth (Gate #50) preserved vascular details and exhibited less noise compared to the input images (b) and (f).

Figure 7 shows the ability of LTDiff++ to enhance spatial resolution by recovering vasculature details from the BFI image at the high gate number of 50. To improve spatial clarity, LTDiff++ was trained on image patches extracted from the two neonatal rat images at Gate #40 (Fig. 7(a) and Fig. 7(e)), with ground truth images at Gate #0 averaged over 100 time-course frames (Fig. 7(d) and Fig. 7(h)). The model was tested on the image patches at Gate #50 (Fig. 7(b) and Fig. 7(f)), where the deeper vessels are less visible. Despite the reduced vascular contrast at greater gates, the reconstructed images (Fig. 7(d) and Fig. 7(h)) successfully restored fine vasculature details.

For neonatal Rat #1, PSNR improved from 10.20 to 13.55 dB, and SSIM increased from 0.25 to 0.35. For neonatal Rat #2, PSNR improved from 10.19 to 13.79 dB, and SSIM increased from 0.249 to 0.35. These results confirm that LTDiff++ effectively recovers brain vascular features at deeper imaging depths, enhancing the visibility of vascular structures.

### 3.5. LTDiff++ enhances temporal resolution of TR-LSCI for CBF images in neonatal rats

**Fig. 8.**
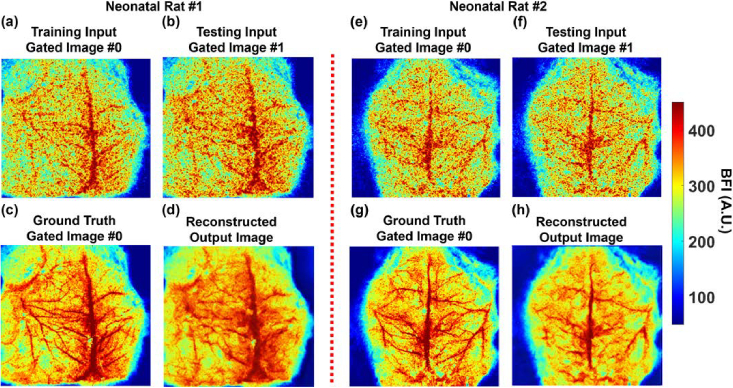
**Temporal resolution enhancement of CBF images in neonatal rats by the LTDiff++ model. (a)** and **(e)** The average BFI image patches taken at Gate #0 for training the LTDiff++ model in Rat #1 and Rat #2 respectively. **(b)** and **(f)** The average BFI image patches taken at Gate #1 for testing the LTDiff++ model in Rat #1 and Rat #2 respectively. The BFI images were obtained by averaging 5 time-course images. **(c)** and **(g)** The average ground truth BFI images taken at Gate #0. The ground truth BFI images were obtained by averaging 100 time-course images to improve SNR. **(d)** and **(h)** The resulting output BFI images with fewer averaging frames (5 frames) preserved vascular details and exhibited less noise compared to the input images (b) and (f), which were averaged over 5 frames.

Reducing the number of averaging time-course BFI images improves temporal resolution, allowing the TR-LSCI system to capture rapid cerebral hemodynamic changes. However, averaging fewer frames decreases SNR, potentially obscuring vascular structures. Figure 8 highlights the capability of LTDiff++ to reduce diffusive noise in BFI images even with fewer averaging frames, thereby enhancing temporal resolution while preserving vascular structure visibility.

The training and testing inputs at Gate #0 (Fig. 8(a) and Fig. 8(e)) and Gate #1 (Fig. 8(b) and Fig. 8(f)) for neonatal Rat #1 and Rat #2, respectively, were generated by averaging 5 time-course BFI images, reducing acquisition time by a factor of 20 compared to averaging 100 images. For both neonatal rats, the average BFI images over 100 frames at Gate #0 were used as the ground truth images (Fig. 8(c) and Fig. 8(g)) for training. Despite the reduced input image quality due to fewer averaging frames, LTDiff++ effectively suppressed diffusive noise and restored cerebral vascular structures (Fig. 8(d) and Fig. 8(h)), achieving results comparable to the ground-truth images averaged over 100 frames.

For neonatal Rat #1, PSNR improved from 11.12 to 15.09 dB, and SSIM increased from 0.22 to 0.29. For neonatal Rat #2, PSNR improved from 6.21 to 10.21 dB, and SSIM increased from 0.069 to 0.12. These results verify that LTDiff++ successfully recovers brain vascular features in BFI images with fewer averaging frames, thereby improving temporal resolution.

## 4. Discussion and conclusions

Frequent assessment of CBF plays a critical role in both clinical care and neuroscience research, particularly for understanding and managing cerebrovascular disorders. While large imaging modalities such as MRI and CT offer snapshots of cerebral anatomy and function, their high cost, immobility, and limited temporal resolution make them impractical for continuous or bedside monitoring. In contrast, diffuse optical imaging techniques have emerged as promising alternatives due to their portability, affordability, and ability to capture real-time hemodynamic changes. Among these, LSCI has gained widespread use in preclinical applications for its ability to provide fast, high-resolution, 2D maps of superficial blood flow. However, conventional LSCI is fundamentally limited by its shallow penetration depth (<1 mm) and inability to distinguish between superficial and deeper vascular layers, which constraints its utility in clinical applications.

This study introduces an advanced TR-LSCI system which stands out for its ability to combine widefield, fast, high-resolution, and depth sensitive imaging of CBF over a wide area of the head. We upgraded the TR-LSCI system, transitioning from the SwissSPAD2 camera to a customized, high-performance SPAD512^2^ camera (Fig. 1). This upgrade streamlined the system setup and imaging workflow, making TR-LSCI more user-friendly and efficient. The SPAD512^2^ camera featured built-in noise corrections and an intuitive software package, making system setup and image acquisition more user-friendly and efficient. Collectively, these advancements significantly enhanced the imaging performance and depth sensitivity of TR-LSCI.

However, TR-LSCI performance has been limited by photon scattering and diffusive noise, particularly at greater depths. Prior deep learning methods for LSCI denoising, such as dilated deep residual learning network (DRSNet) [27], lightweight denoising speckle contrast image generative adversarial network (LDSCI-GAN) [28], and spatial frequency cycle-consistent generative adversarial network (SF-CycleGAN) [29], were developed for conventional LSCI systems under continuous-wave illumination. These approaches operate on surface-level 2D contrast images and assume spatially homogeneous, depth-invariant speckle noise. However, such assumptions and architectures are not directly applicable to TR-LSCI, where depth-dependent photon statistics, gate-varying SNR, and diffusive noise complicate image fidelity particularly at greater depths.

To address these challenges, we developed LTDiff++, the first latent diffusion model tailored for TR-LSCI (Fig. 2). LTDiff++ incorporates a UNet++ encoder–decoder with intermediate convolutional layers which gradually refine and align features before fusion to better preserve multi-scale vascular features, which are highly susceptible to degradation in low-SNR, depth-resolved imaging [30]. Unlike conventional denoising methods that operate in image space, LTDiff++ performs conditional denoising diffusion in latent space, harmonizing depth-varying features by modeling the full distribution of clean BFI representations [20,21]. This latent-space formulation improves generalizability and reduces risks of over-smoothing or artifacts which are the common limitations of CNN and GAN based models trained with pixel-wise losses [18,31].

LTDiff++ further employs a structured three-phase training strategy to stabilize optimization across encoder, diffusion, and decoder components. A hybrid loss function provides dual supervision in both latent and image domains, encouraging anatomical fidelity and radiomic texture preservation across varying depths. Together, these architectural and algorithmic innovations enable LTDiff++ to enhance image quality at deeper gates, reduce the need for heavy frame averaging, and improve robustness in depth-resolved TR-LSCI applications.

To ensure optimal training of LTDiff++, TR-LSCI data from both head-simulating phantoms and neonatal rats were preprocessed into high-overlap image patches, enabling the model to learn localized features while maintaining global structural consistency. For the phantom data, image patches were extracted from separate regions of the UK-logo phantom to create non-overlapping training and testing datasets. For neonatal rat data, the entire head BFI images were segmented into overlapping patches as the model input (Fig. 3). To encourage generalizability, patches were sampled from both rats and covered diverse anatomical regions across the brain. We selected a patch size of 64 × 64 pixels as it provided an optimal balance between capturing sufficient local vascular structures and ensuring computational efficiency during training. This patch size was large enough to encompass meaningful anatomical features such as vessel segments and local flow patterns, while remaining small enough to facilitate efficient memory usage and faster convergence of the LTDiff++ model, particularly given the complexity of the UNet++ encoder-decoder and latent diffusion components. While leave-one-rat-out validation was not feasible due to the limited datasets (n = 2), the model consistently enhanced vascular structures across rats and time points. Although trained using 64 × 64 patches, the model was evaluated on non-overlapping patches covering the entire brain field of view, including areas not seen during training. The consistent enhancement of vascular structures and improvements in PSNR and SSIM across unseen regions demonstrate that the chosen patch size effectively supports both spatial generalization and high-quality image enhancement.

We first demonstrated the depth-resolved imaging capabilities of TR-LSCI using a 3D-printed head-simulating phantom embedded with a flow channel shaped like the UK logo and 1-mm top layer thickness (Fig. 4). Flow contrast images captured at increasing gate numbers from #40 to #120 showed progressively deeper photon penetration but also increasing photon diffusive noise, which limited the spatial resolution at later gates. Notably, a strong UK logo signal emerged around Gate #107, motivating its selection as a representative noisy gate for image enhancement. Additionally, Gate #40 was included to explore the model's performance at a shallower depth, providing two distinct input conditions for denoising evaluation in the phantom data (Fig. 5). LTDiff++ effectively enhanced the image quality of phantom data acquired at both Gate #107 and Gate #40, recovering structural details obscured by diffusive noise. This was evident in the model reconstructed outputs, which displayed significantly improved PSNR and SSIM scores compared to the unprocessed input images.

In vivo imaging of neonatal rats (Fig. 6) further demonstrated the depth sensitivity of the TR-LSCI system. As the gate number increased from #0 to #50, deeper cerebral vessels became less visible due to photon scattering and intensity attenuation. These findings were consistent across both rats and confirmed the expected degradation in signal quality with increasing depth. LTDiff++ was then applied to improve spatial resolution in these deep gates (Fig. 7). The model was trained using image patches at Gate #40 and tested on Gate #50, with high-quality ground truth derived from Gate #0 averaged over 100 time-course images.

Importantly, in vivo imaging was performed with the skull intact to preserve physiological integrity, meaning LTDiff++ was evaluated under realistic conditions that include static scattering from bone. Despite the additional photon attenuation and noise introduced by the skull, the model effectively restored vascular structures even at greater depths (e.g., Gate #50), demonstrating its robustness to nonuniform photon distributions and bulk tissue scattering. This robustness is especially relevant for translational applications where imaging through intact skulls is often unavoidable. These results validate the model’s ability to suppress diffusive noise and recover spatial details lost in TR-LSCI images at deeper depths. Still, future work will involve systematic testing across new subjects and brain regions to further validate cross-sample and spatial generalizability.

Temporal resolution enhancement was also evaluated by training LTDiff++ on time-course images. Only five BFI frames were averaged at Gates #0 and #1 for training and testing respectively (Fig. 8), in contrast to 100 in earlier data processing. Despite the drastic reduction in frame averaging (from 100 to 5), the model successfully reconstructed BFI images with quality comparable to the ground truth with 100-frame averaging. This capability could enable faster TR-LSCI imaging without sacrificing image fidelity, critical for tracking rapid hemodynamic events. Beyond acquisition time reduction, we also evaluated the model's inference speed to assess its suitability for real-time applications. When running on an NVIDIA A100 GPU (80GB), LTDiff++ processed a 64 × 64 TR-LSCI frame in approximately 0.47 seconds. While not yet real-time, this inference time is substantially faster than traditional averaging (100-frame) approaches and competitive with other deep learning models used in biomedical imaging [27]. Future efforts will focus on optimizing network architecture, using lightweight backbones, and leveraging hardware accelerators (e.g., TensorRT or FPGAs) to further reduce latency and move toward real-time TR-LSCI applications.

An observation in this study was the inconsistency in the optimal gate numbers to obtain the best quality of BFI images between the phantom and animal experiments (Fig. 4 and Fig. 6). While phantom results showed the best BFI image around Gate #107 (Fig. 4), in-vivo vascular features were best observed around Gates #0-40 (Fig. 6). This discrepancy is due in part to the differences in the optical properties and geometry between the fabricated head-simulating phantom and neonatal rat heads. Reported average optical properties in rat brains indicate that the μ_a_ in gray matter (∼5.4 cm^−1^) is higher than in white matter (∼0.29 cm^−1^), while the μ_s_’ in gray matter (16.7 cm^−1^) is lower than in white matter (∼21.3 cm^−1^) [32]. In contrast, the head-simulating phantom was constructed with homogeneous optical properties (μ_a_ = 0.03 cm^−1^ and μ_s_’ = 9 cm^−1^), leading to differences in photon transport behavior and effective gate delays. Additionally, the geometries of the head-simulating phantom and neonatal rat heads are not identical.

Another contributing factor to the gate number discrepancy was the hardware transition from SwissSPAD2 to SPAD512^2^. Differences in offset times stemming from cable-induced delays, FPGA processing latency, and the delay between the two halves of the sensor resulted in shifts in the gate offset (i.e., t_0_, the delay from the sync pulse). As a result, each experiment required a different t_0_ setting, contributing to the observed inconsistencies in the optimal gate numbers to obtain the best quality of BFI images between the phantom and animal experiments.

While a detailed sensitivity analysis and formal statistical significance testing were not conducted due to the limited sample size (n = 1 phantom, n = 2 neonatal rats) and absence of pathological variation, the consistent improvements in PSNR and SSIM across different gates, subjects, and imaging conditions suggest that LTDiff++ maintains robustness under moderate inter-sample variability. Since a single LTDiff++ model was trained and applied across all test cases, comparative statistical analyses (e.g., paired t-tests or ANOVA) would not yield meaningful or interpretable results. This study is therefore positioned as a proof-of-concept to demonstrate the feasibility and performance of latent diffusion modeling for TR-LSCI enhancement under realistic imaging conditions. Future studies will incorporate larger cohorts, disease models, and tunable optical phantoms to enable quantitative assessment of sensitivity to tissue optical properties and geometry, as well as rigorous statistical evaluations to further validate model generalizability and reproducibility.

In conclusion, this study presents a comprehensive advancement in TR-LSCI for depth-sensitivity, high-resolution, and fast imaging of CBF. By integrating a customized, user-friendly, and high-performance SPAD512^2^ camera and LTDiff++ deep learning framework for diffusive noise reduction, the upgraded TR-LSCI system offers significant improvements in both spatial and temporal resolution. We validated the method using both head-simulating phantom and neonatal rat data, demonstrating its effectiveness in suppressing diffusive noise and recovering structure and vasculature at deeper imaging depths. Moreover, in-vivo studies showed that LTDiff++ preserved image quality with only 5-frame averaging, effectively reducing acquisition time by 20-fold compared to the traditional 100-frame averaging method without deep learning enhancement. Looking ahead, additional enhancements are planned through the deployment of a customized SPAD512^2^ camera featuring integrated microlenses, resulting in a fill factor exceeding 50%. This modification is expected to improve photon collection efficiency by 3- to 4-fold, substantially increasing imaging sensitivity and depth sensitivity for future TR-LSCI studies. Future studies will aim to generalize the LTDiff++ model across different tissue types and extend this framework to multi-wavelength TR-LSCI for simultaneous assessment of blood flow and oxygenation. This upgraded technique paves the way for noninvasive, high-performance neuroimaging in both preclinical and clinical settings.

## Data Availability

Data underlying the results presented in this paper are not publicly available at this time but may be obtained from the authors upon reasonable request.

## References

[1] Fantini S. Sassaroli A. Tgavalekos K. T. et al., “Cerebral blood flow and autoregulation: current measurement techniques and prospects for noninvasive optical methods,” Neurophotonics 3(3), 031411 (2016). 10.1117/1.NPh.3.3.031411 27403447 PMC4914489

[2] Buckley E. M. Parthasarathy A. B. Grant P. Ellen et al., “Diffuse correlation spectroscopy for measurement of cerebral blood flow: future prospects,” Neurophotonics 1(1), 011009 (2014). 10.1117/1.NPh.1.1.011009 25593978 PMC4292799

[3] Silverman A. Petersen N.H., Physiology, cerebral autoregulation. 2020. 31985976

[4] Willie C. K. Tzeng Y.-C. Fisher J. A. et al., “Integrative regulation of human brain blood flow,” The Journal of physiology 592(5), 841–859 (2014). 10.1113/jphysiol.2013.268953 24396059 PMC3948549

[5] Grade M. Hernandez Tamames J. A. Pizzini F. B. et al., “A neuroradiologist’s guide to arterial spin labeling MRI in clinical practice,” Neuroradiology 57(12), 1181–1202 (2015). 10.1007/s00234-015-1571-z 26351201 PMC4648972

[6] Wierenga C. E. Hays C. C. Zlatar Z. Z., “Cerebral blood flow measured by arterial spin labeling MRI as a preclinical marker of Alzheimer's disease,” Journal of Alzheimer's disease 42(s4), S411–S419 (2014). 10.3233/JAD-141467 PMC5279221 25159672

[7] Cenic A. Nabavi D. G. Craen R. A. et al., “Dynamic CT measurement of cerebral blood flow: a validation study,” American Journal of Neuroradiology 20(1), 63–73 (1999). 9974059

[8] Chugani H. T., “A critical period of brain development: studies of cerebral glucose utilization with PET,” Prev. Med. 27(2), 184–188 (1998). 10.1006/pmed.1998.0274 9578992

[9] Ibaraki M. Miura S. Shimosegawa E. et al., “Quantification of cerebral blood flow and oxygen metabolism with 3-dimensional PET and 15O: validation by comparison with 2-dimensional PET,” J. Nucl. Med. 49(1), 50–59 (2008). 10.2967/jnumed.107.044008 18077532

[10] Markwalder L. Richards L. M. Khan F. et al., “In vivo laser speckle contrast imaging of microvascular blood perfusion using a chip-on-tip camera,” iScience 27(3), 109077 (2024). 10.1016/j.isci.2024.109077 38375226 PMC10875563

[11] Richards L. M. Kazmi S. M. Shams Davis J. L. et al., “Low-cost laser speckle contrast imaging of blood flow using a webcam,” Biomed. Opt. Express 4(10), 2269–2283 (2013). 10.1364/BOE.4.002269 24156082 PMC3799684

[12] Vaz P. G. Humeau-Heurtier A. Figueiras E. et al., “Laser speckle imaging to monitor microvascular blood flow: a review,” IEEE Rev. Biomed. Eng. 9, 106–120 (2016). 10.1109/RBME.2016.2532598 26929060

[13] Boas D. A. Dunn A. K., “Laser speckle contrast imaging in biomedical optics,” J. Biomed. Opt. 15(1), 011109 (2010). 10.1117/1.3285504 20210435 PMC2816990

[14] Senarathna J. Rege A. Li N. et al., “Laser speckle contrast imaging: theory, instrumentation and applications,” IEEE Rev. Biomed. Eng. 6, 99–110 (2013). 10.1109/RBME.2013.2243140 23372086

[15] Fathi F. Fathi F. Mohtasebi M. et al., “Laser speckle contrast imaging of cerebral blood flow using picosecond pulsed laser illumination,” in *Multiscale Imaging and Spectroscopy III* . 2022. SPIE.

[16] Fathi F. Yu G. Singh D. et al., “Time-resolved laser speckle contrast imaging (TR-LSCI) of cerebral blood flow,” IEEE Trans. Med. Imaging 44(3), 1206 (2024). 10.1109/TMI.2024.3486084 PMC11995863 39446549

[17] Yu G. Jain A. Abbeel P. et al., “Multi-wavelength time-resolved laser speckle contrast imaging (mtr-lsci) of tissue hemodynamics and metabolism,” 2024, Google Patents.

[18] Ho J. Jain A. Abbeel P., “Denoising diffusion probabilistic models,” Advances in neural information processing systems 33, 6840–6851 (2020). 10.5555/3495724.3496298

[19] Nichol A.Q. Dhariwal P., “Improved denoising diffusion probabilistic models,” in International conference on machine learning. 2021. PMLR.

[20] Sadia R.T. Zhang J. Chen J., “Multiscale Latent Diffusion Model for Enhanced Feature Extraction from Medical Images,” arXiv (2024). 10.48550/arXiv.2410.04000

[21] Selim M. Zhang J. Fathi F. et al., “Latent Diffusion Model for Medical Image Standardization and Enhancement,” arXiv (2023). 10.48550/arXiv.2310.05237 PMC10785850 38222387

[22] Fan Y. Huijben E.M. Pluim J.P. et al., “A survey of emerging applications of diffusion probabilistic models in MRI,” Meta-Radiology 2(2), 100082 (2024).

[23] Gong K. Abouei E. Wynne J. et al., “PET image denoising based on denoising diffusion probabilistic model,” Eur. J. Nucl. Med. Mol. Imaging 51(2), 358–368 (2024). 10.1007/s00259-023-06417-8 37787849 PMC10958486

[24] Huijben E. M. Pluim J. P. van Eijnatten M. A., “Denoising diffusion probabilistic models for addressing data limitations in chest X-ray classification,” Informatics in Medicine Unlocked 50, 101575 (2024). 10.1016/j.imu.2024.101575

[25] Pan S. Lu J. Zhu X. et al., “Synthetic CT generation from MRI using 3D transformer-based denoising diffusion model,” Med. Phys. 51(4), 2538–2548 (2024). 10.1002/mp.16847 38011588 PMC10994752

[26] Kotyan S. Ueda T. Vargas D.V., “k* Distribution: Evaluating the Latent Space of Deep Neural Networks Using Local Neighborhood Analysis,”IEEE Transactions on Neural Networks and Learning Systems, 2024. 10.1109/TNNLS.2024.3446509 39283786

[27] Cheng W. Li D. Hu Z. et al., “Dilated residual learning with skip connections for real-time denoising of laser speckle imaging of blood flow in a log-transformed domain,” IEEE Trans. Med. Imaging 39(5), 1582–1593 (2020). 10.1109/TMI.2019.2953626 31725373

[28] Sang X. Yip S.S. Niu L. et al., “Lightweight denoising speckle contrast image GAN for real-time denoising of laser speckle imaging of blood flow,” Biomed. Opt. Express 16(3), 1118–1142 (2025). 10.1364/BOE.545628 40109526 PMC11919355

[29] Du Y. Li D. Hu S. et al., “Dual-Channel in Spatial-Frequency Domain CycleGAN for perceptual enhancement of transcranial cortical vascular structure and function,” Comput. Biol. Med. 173, 108377 (2024). 10.1016/j.compbiomed.2024.108377 38569233

[30] Zhou Z. Tanikawa Y. Okada E. et al., “Unet++: A nested u-net architecture for medical image segmentation,” in Deep learning in medical image analysis and multimodal learning for clinical decision support, 4th international workshop, DLMIA 2018, and 8th international workshop, ML-CDS 2018, held in conjunction with MICCAI 2018, Granada, Spain, September 20, 2018, proceedings 4. 2018. Springer. 10.1007/978-3-030-00889-5_1 PMC7329239 32613207

[31] Yip S. S. Aerts H.J., “Applications and limitations of radiomics,” Phys. Med. Biol. 61(13), R150–R166 (2016). 10.1088/0031-9155/61/13/R150 27269645 PMC4927328

[32] Hoshi Y. Tanikawa Y. Okada E. et al., “In situ estimation of optical properties of rat and monkey brains using femtosecond time-resolved measurements,” Sci. Rep. 9(1), 9165 (2019). 10.1038/s41598-019-45736-5 31235830 PMC6591507

